# Predicting Long non-coding RNAs through feature ensemble learning

**DOI:** 10.1186/s12864-020-07237-y

**Published:** 2020-12-17

**Authors:** Yanzhen Xu, Xiaohan Zhao, Shuai Liu, Wen Zhang

**Affiliations:** College of Informatics, Huazhong Agricultural University, Wuhan, 430070 China

**Keywords:** lncRNA prediction, Attention mechanism, Feature ensemble learning

## Abstract

**Background:**

Many transcripts have been generated due to the development of sequencing technologies, and lncRNA is an important type of transcript. Predicting lncRNAs from transcripts is a challenging and important task. Traditional experimental lncRNA prediction methods are time-consuming and labor-intensive. Efficient computational methods for lncRNA prediction are in demand.

**Results:**

In this paper, we propose two lncRNA prediction methods based on feature ensemble learning strategies named LncPred-IEL and LncPred-ANEL. Specifically, we encode sequences into six different types of features including transcript-specified features and general sequence-derived features. Then we consider two feature ensemble strategies to utilize and integrate the information in different feature types, the iterative ensemble learning (IEL) and the attention network ensemble learning (ANEL). IEL employs a supervised iterative way to ensemble base predictors built on six different types of features. ANEL introduces an attention mechanism-based deep learning model to ensemble features by adaptively learning the weight of individual feature types. Experiments demonstrate that both LncPred-IEL and LncPred-ANEL can effectively separate lncRNAs and other transcripts in feature space. Moreover, comparison experiments demonstrate that LncPred-IEL and LncPred-ANEL outperform several state-of-the-art methods when evaluated by 5-fold cross-validation. Both methods have good performances in cross-species lncRNA prediction.

**Conclusions:**

LncPred-IEL and LncPred-ANEL are promising lncRNA prediction tools that can effectively utilize and integrate the information in different types of features.

## Background

In the last few decades, due to the development of high-throughput sequencing technologies, a great number of transcripts have been generated [1]. Transcripts are a combination of DNA translation products, including mRNAs, tRNAs, rRNAs, and non-coding RNAs (ncRNAs). NcRNAs are a class of RNAs that do not encode any protein, and lncRNAs (long non-coding RNAs) are ncRNAs with lengths exceeding 200 nucleotides (nt). Although lncRNAs are not translated into proteins, they are of great significance in various cellular development progresses, such as gene expression/regulation [2], gene silencing [3], RNA modification [4]. More importantly, lncRNAs have been proved to be associated with many diseases, for instance, DD3 is related to prostate cancer [5] and BACE1-AS is related to Alzheimer’s disease [6]. Predicting lncRNAs from transcripts is important to the downstream biological function analysis. However, traditional experimental methods for lncRNA identification are time-consuming and labor-intensive, thus cannot perform lncRNA prediction when dealing with a massive number of transcripts. With the increasing number of transcripts, efficient computational methods especially machine learning methods for lncRNA prediction are demanded. Researchers have proposed many machine learning methods for lncRNA prediction in the last few years. These lncRNA prediction methods can be categorized into three major types, binary classifier-based methods, deep learning-based methods, and ensemble learning-based methods.

The binary classifier-based methods consider lncRNA prediction as a binary classification task of two types of transcripts: lncRNAs and protein-coding transcripts (PCTs). Such methods make use of different features, such as codon-related features, ORF-related features, GC-related features, coding sequence-related features, and structure-related features, then design classifiers to build prediction models. Support vector machine (SVM) [7] is a supervised learning model using associated learning algorithms to analyze the data, which is the most commonly used classifier for lncRNA prediction. SVM [7] is adopted in lncRNA prediction methods such as CPC [8], CNCI [9], PLEK [10], lncRScan-SVM [11], CPC2 [12] Longdist [13], and CPPred [14]. Random forest (RF) [15] uses the bagging strategy to build trees and then constructs an uncorrelated forest of trees to make predictions. RF is also commonly adopted in the lncRNA prediction models, such as LncRNApred [16], LncRNA-ID [17], COME [18], and FEElnc [19]. Logistic regression (LR) [20] is a statistical algorithm used to model the probability of a certain class, which is used in Tradigo et al.’s work [21] and CPAT [22].

Recently, deep learning architecture shows the great ability of fitting complex functions and achieves high performance in bioinformatics [23], so it is also applied to lncRNA prediction. For example, lncRNA-MFDL [24] constructs a powerful lncRNA predictor by fusing multiple features based on the deep learning algorithm. lncRNAnet [25] uses the recurrent neural network (RNN) for RNA sequence modeling and the convolutional neural network (CNN) for detecting stop codons to better identify lncRNAs. LncADeep [26] integrates intrinsic and homology features to construct a deep belief network. DeepLNC [27] uses k-mer patterns to construct a deep neural network (DNN) for the identification of lncRNAs. To enhance the generalization ability and performance of models further, several ensemble learning-based methods have been developed. Ensemble learning methods combine multiple classifiers to obtain better prediction performance [28], and bagging, boosting and voting are three common ensemble learning strategies for combining multiple classifiers. TLCLnc [29] is a two-layer structured ensemble learning model. The first layer of TLCLnc is the stacking of base SVM predictors which takes a disjoint set of features as inputs, and the second layer is the naïve Bayes classifier. Simopoulos et al. [30] put forward a plant lncRNA prediction method based on the stochastic gradient boosting of random forest classifiers. DeepCPP [31] considers nucleotide bias information and minimum distribution similarity feature selection to construct a DNN model and calculate the coding potential of transcripts.

Existing methods make great progress in lncRNA prediction; however, we want to stress two aspects for improvements. On the one hand, for feature usage, most existing lncRNA prediction methods often utilize features specifically used for biological transcripts [8, 18, 22], which we called transcript-specified features in the following sections. However, there are some common statistical features of nucleotide sequences, which we named general sequence-derived features in the following discussion, are rarely adopted. For example, the general sequence-derived feature CTD mentioned in Liu’s work [14] is rarely adopted in existing lncRNA prediction methods to our knowledge. On the other hand, for model construction, although ensemble learning models and deep learning models have been used in lncRNA prediction methods, existing models lack consideration for the intricate interactions between different types of features. As discussed above, the ensemble learning-based lncRNA prediction methods usually adopt simple boosting or stacking ensemble strategies, and deep learning-based methods usually utilize basic DNN, RNN, and CNN models. These model designs can cause the oversights of useful information when integrating the features to build the prediction model. Thus, flexible and robust ensemble learning and deep learning model constructions are still demanded for exploiting the information in features to better facilitate lncRNA prediction.

In this study, we propose two lncRNA prediction methods based on feature ensemble learning strategies, namely LncPred-IEL and LncPred-ANEL. First, we extract transcript-specified features and general sequence-derived features from transcripts. Second, we consider two feature ensemble strategies to integrate the information from different feature types, namely iterative ensemble learning (IEL) and attention network ensemble learning (ANEL). In the previous study, LncPred-IEL [32] builds base predictors based on different types of features and employs a supervised iterative way to combine base predictors and build ensemble models. As the extension of LncPred-IEL [32], we propose a novel lncRNA prediction method named LncPred-ANEL, which adopts a deep neural network with the attention mechanism [33] to ensemble different types of features by adaptively learning the weight of individual feature types.

After model construction, we conduct experiments to test the performances of the proposed models. We adopt LargeVis [34] to visualize the feature vectors before and after feature ensemble, and results demonstrate that IEL and ANEL can differentiate lncRNAs from other transcripts in feature space, which means the feature ensemble strategies can effectively exploit and integrate the information in different types of features. Then we compare LncPred-IEL and LncPred-ANEL with four state-of-the-art methods and results have shown that both methods have better performances on evaluation metrics. Furthermore, we test the models on the cross-species datasets and obtain good results, indicating the models have good generalization ability.

## Results and discussion

### Evaluation metrics

We use 5-fold cross-validation (5-CV) to evaluate the prediction models. To perform 5-CV, the datasets are equally split into 5 subsets. One subset is used as the testing set, 20% of the remaining four subsets are used as the validation set and 80% are used as the training set. In a fold of 5-CV, we train the models on the training set, and determine the optimal model parameters on the validation set, then utilize the model to make predictions on the testing set. This training-validation-testing process is repeated 5 times until each subset has been used for testing. The performances of prediction models are evaluated by several commonly used metrics such as sensitivity (SN), specificity (SP), accuracy (ACC) score, and the area under curve (AUC), given by

$$ \mathrm{SN}=\frac{\mathrm{TP}}{\mathrm{TP}+\mathrm{FN}} $$

$$ \mathrm{SP}=\frac{\mathrm{TN}}{\mathrm{TN}+\mathrm{FP}} $$

$$ \mathrm{ACC}=\frac{\mathrm{TP}+\mathrm{TN}}{\mathrm{TP}+\mathrm{TN}+\mathrm{FP}+\mathrm{FN}} $$

where true positive (TP) is the number of true positive instances predicted to be positive; true negative (TN) is the number of true negative instances predicted to be negative; false positive (FP) is the number of negative instances predicted to be positive; false negative (FN) is the number of positive instances predicted to be negative. The receiver operating characteristic (ROC) curve is plotted by using the false positive rate (1-SP) against SN for different cutoff thresholds and the AUC score is the area under the ROC curve. We take AUC as the primary evaluation metric because it assesses the performances of prediction models regardless of any threshold.

### Parameter settings for features

As shown in Table 2, several general sequence-derived features have parameters. It is critical to determine the parameters for these features because the parameter settings will influence the performances of prediction models.

The feature *k*-mer has a parameter *k*, we consider *k* = 1, 2, 3, 4, 5 respectively, and merge all of them as the spectrum profile. The same setting is adopted for the reverse complement *k*-mer profile.

The mismatch profile has two parameters *k* and *m*. We adopt the same parameter setting as the spectrum profile for *k*. The parameter *m* means the maximum mismatch tolerance, in this study, we suppose that *m* does not exceed one-third of the length of *k*-mer. So, we choose the (3, 1)-mismatch profile, the (4, 1)-mismatch profile, and the (5, 1)-mismatch profile and merge them to obtain the mismatch profile.

The pseudo nucleotide composition features PseDNC, PC-PseDNC-General, PC-PseTNC-General, SC-PseDNC-General, and SC-PseTNC-General have two parameters (*λ*, *w*), where 0.1 ≤ *w* ≤ 0.9 and *λ* is the highest counted rank of correlation. For PseDNC, PC-PseDNC-General, and SC-PseDNC-General, 1 ≤ *λ* ≤ *L* − 2; for PC-PseTNC-General and SC-PseTNC-General, 1 ≤ *λ* ≤ *L* − 3. *L* is the shortest length of transcripts and *L* = 9 in the main datasets. To determine the best parameter combinations, we use a grid search strategy and build RF prediction models on the balanced CPPred [14] Human dataset with different combinations of the two parameters in the above ranges. The RF prediction models achieve the highest AUC score when using parameters (7, 0.5), (7, 0.7), (6, 0.7), (7, 0.1), and (6, 0.1) for PseDNC, PC-PseDNC-General, PC-PseTNC-General, SC-PseDNC-General, and SC-PseTNC-General respectively. We adopt the above settings for pseudo nucleotide composition features.

The auto-cross covariance features DACC and TACC have a parameter *lag* and 1 ≤ *lag* ≤ *L* − 2. To determine the parameter *lag*, we build RF prediction models on the balanced CPPred [14] Human dataset with different *lag*. We obtain the highest AUC score when *lag* = 7, so we set *lag* to 7 for auto-cross covariance features.

Therefore, all parameters are determined for features, then we encode transcripts into feature vectors.

### Convergence analysis of proposed methods

LncPred-IEL uses an iterative way to build the prediction model, and LncPred-ANEL trains the prediction model by optimizing the network. In this section, we want to investigate the training processes of LncPred-IEL and LncPred-ANEL on the main datasets of Human and Mouse to analyze the convergence of proposed methods.

**Fig. 1:**
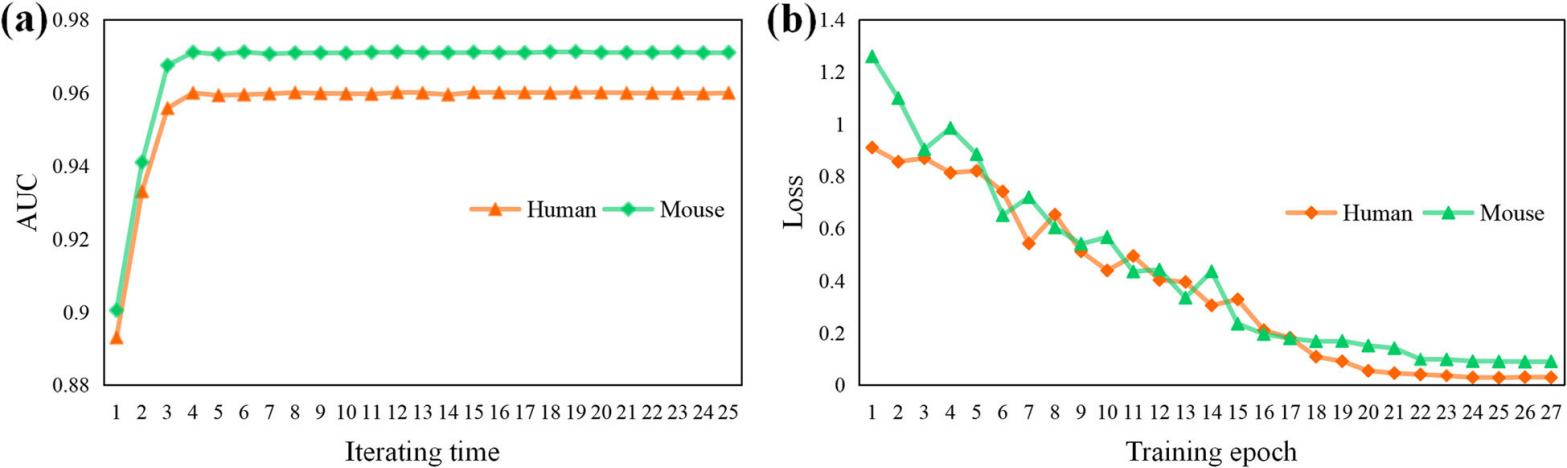
Training processes of LncPred-IEL and LncPred-ANEL. **a** AUC scores of LncPred-IEL models in each iterating time. **b** Loss scores of LncPred-ANEL models in each training epoch

For both models, the training process will converge until there are no significant changes in performance. To investigate the convergence, we record the 5-CV performances of LncPred-IEL and LncPred-ANEL in the training processes. From Fig. 1a, we can see that LncPred-IEL produces the AUC scores of 0.9601 and 0.9709 in the 8th iterating time on the main datasets of Human and Mouse respectively. From Fig. 1b, we can see that LncPred-ANEL produces the loss scores of 0.0356 and 0.0982 in the 23rd training epoch on the main datasets of Human and Mouse respectively. No significant changes in performance metrics are observed afterward. In general, the performances of both models increase as the training processes continue and converge after the training rounds above. Results show that both models can gradually improve lncRNA prediction performances with the training process and then converge to stable performance values.

We point out that both LncPred-IEL and LncPred-ANEL reach convergence very fast. However, due to the availability of GPU acceleration of neural network and less training parameters, the time consumption of LncPred-ANEL is 729 s, which is less than a third of LncPred-IEL of 2319 s when tested on our workstation (Intel(R) Xeon(R) Gold 6146 CPU, NVIDIA 1080 Ti GPU and 128G RAM). In the following experiments, the iterative round of LncPred-IEL is set to 18 and the training epoch of LncPred-ANEL is set to 23.

### Feature ensemble enhance performance

In this section, we will investigate how the feature ensemble strategies enhance performance by utilizing and integrating different types of features to make predictions. We take the results on the main Mouse dataset for example.

**Fig. 2:**
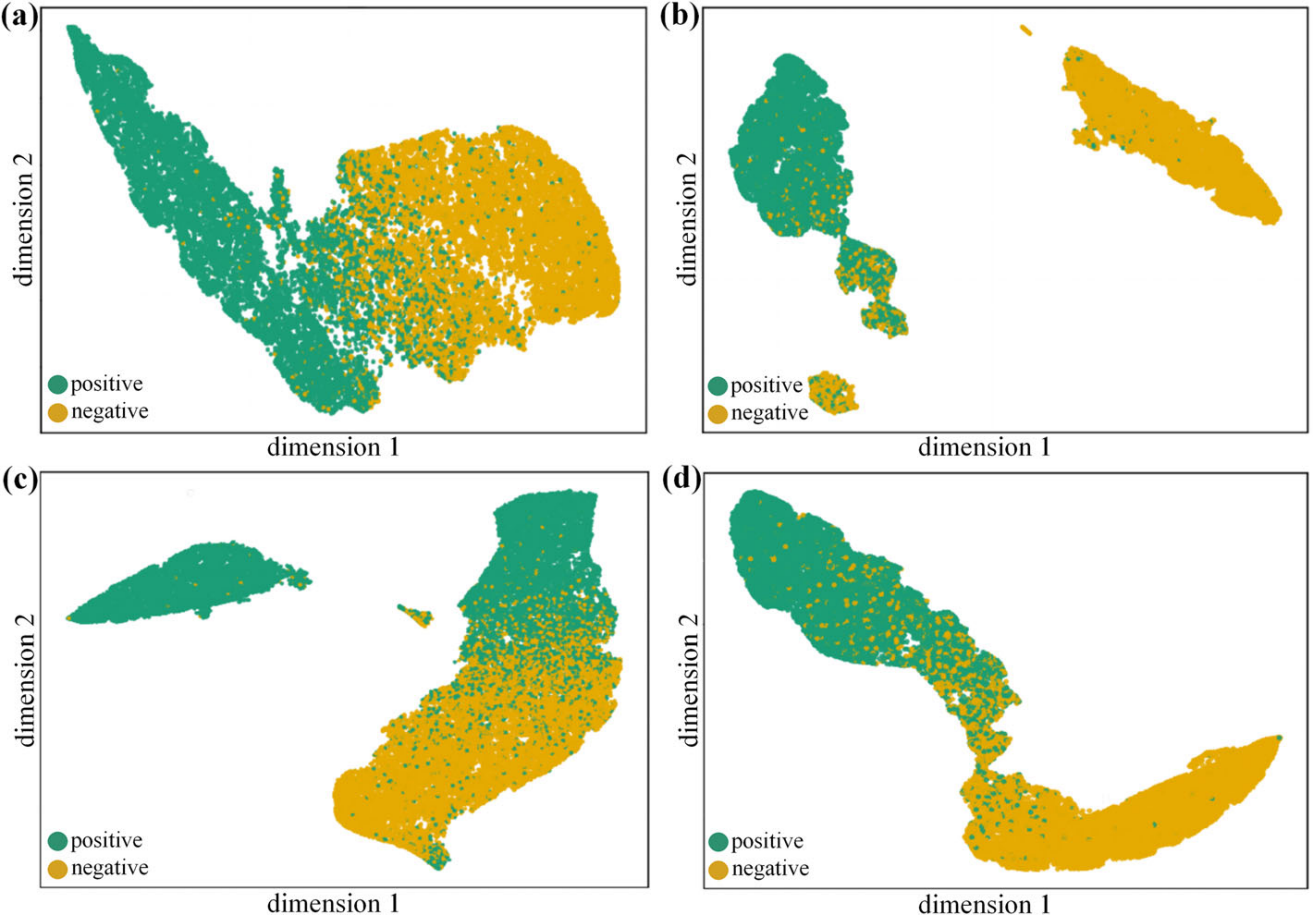
LargeVis visualization of feature vectors. **a** and **b** are the visualizations of feature vectors before and after feature ensemble of LncPred-IEL respectively. **c** and **d** are the visualizations of feature vectors before and after feature ensemble of LncPred-ANEL respectively

We utilize a visualization tool for large-scale and high-dimensional data called LargeVis [34], which uses the data to construct an accurate approximated K-nearest neighbor graph and then demonstrates the graph in a low-dimensional space. We take the feature vectors before and after feature ensemble and utilize LargeVis to display the distribution of positive instances and negative instances in the 2-dimensional feature space, which is shown in Fig. 2. Specifically, for LncPred-IEL, we take the 6-dimensional input vectors in the first iterating time and the 24-dimensional input vectors in the 18th iterating time, which are visualized in Fig. 2a and b respectively. For LncPred-ANEL, we take the feature embedding vectors on the first training epoch and the attention embedding vectors in the 22nd epoch where loss is very low, which are visualized in Fig. 2c and d respectively.

As we can see in Fig. 2a and c, positive instances and negative instances are not classified by the initial feature vectors, while in Fig. 2b and d, positive instances are separated from negative instances after the feature ensemble. We can draw three conclusions from the results, first, the performances of the two methods enhance with the training processes. Second, both methods can extract useful information from the six types of features to facilitate prediction. Third, both feature ensemble learning methods can effectively integrate six types of features based on interactions between different feature types to distinguish lncRNAs from PCTs. Specifically, the iterative feature ensemble process in LncPred-IEL and the attention mechanism to selectively concentrate on important feature types in LncPred-ANEL can better facilitate the prediction results.

### Comparison with state-of-the-art methods

In this section, we compare LncPred-IEL and LncPred-ANEL with several state-of-the-art methods including CPAT [22], CPC2 [12], Longdist [13], and CPPred [14]. Those lncRNA prediction methods reported good performances. We adopt the default parameter setting described in the original paper for state-of-the-art methods. All prediction methods are evaluated using 5-CV.

**Fig. 3:**
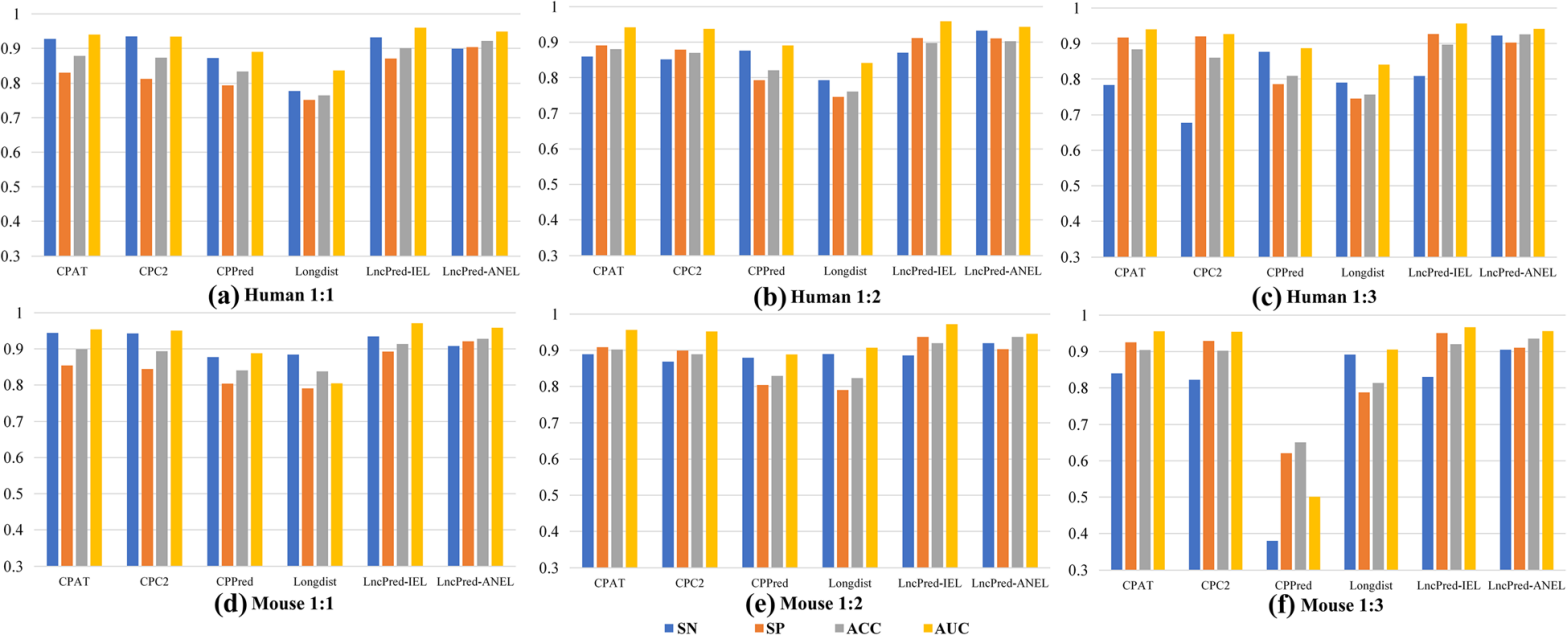
LncRNA prediction model performances on balanced and imbalanced datasets of Human and Mouse respectively

We compare LncPred-IEL and LncPred-ANEL with other methods using the main datasets of Human and Mouse, which are balanced datasets containing the same number of lncRNAs and PCTs. The results are shown in Fig. 3a and d, LncPred-IEL achieves the highest AUC scores and LncPred-ANEL has the second-highest AUC scores. And we point out that LncPred-ANEL has the highest ACC scores indicating that LncPred-ANEL can accurately predict lncRNAs from PCTs. LncPred-ANEL also has the highest SP scores, suggesting it could reduce false-positive predictions. As far as we are concerned, there are two reasons why LncPred-IEL and LncPred-ANEL outperform others. First, both methods utilize general sequence-derived features, bringing common statistical information of nucleotide sequences to the prediction. Second, LncPred-IEL and LncPred-ANEL are flexible feature ensemble methods, which can extract and integrate information from different types of features to enhance lncRNA prediction performances.

Furthermore, we also test the methods on imbalanced datasets of Human and Mouse, which have 1:2 and 1:3 ratio of the number of positive instances to negative instances. As we can see in Fig. 3b, c, e, and f, LncPred-IEL always has the highest AUC scores and LncPred-ANEL always has the second-highest AUC scores, suggesting that both methods are robust on imbalanced datasets. Noteworthy, although SN scores of most methods decline because the prediction results of models trained on imbalanced datasets favor the majority type i.e. PCTs. LncPred-ANEL always has the highest SN scores, indicating it can effectively distinguish lncRNAs and can capture some intrinsic information from features that makes lncRNAs different from PCTs.

### Cross-species prediction

In this section, we test the ability of LncPred-IEL and LncPred-ANEL to perform cross-species lncRNA prediction. We train both models on the main datasets of Human and Mouse, and then make predictions for transcripts from CPPred Fruit Fly and Zebrafish datasets, detailed description of the datasets can be found in Table 1.

**Table 1:** Summary of the datasets

Description	Species	# Positive	# Negative
Main datasets	Human	24,162	24,162
	Mouse	27,595	27,595
CPPred datasets	Human	23,384	23,384
	Mouse	15,345	15,345
	Fruit Fly	2775	17,399
	Zebrafish	6840	15,534

**Fig. 4:**
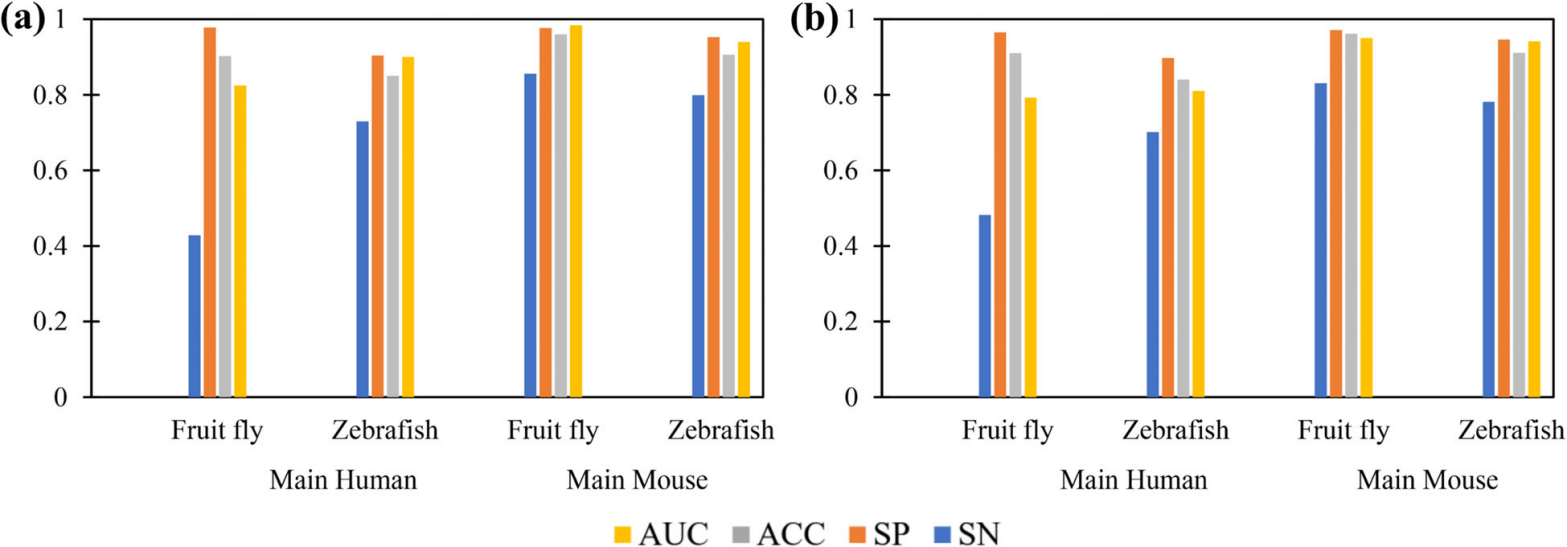
Cross-species prediction results of LncPred-IEL (**a**) and LncPred-ANEL (**b**)

Figure 4 shows that LncPred-IEL and LncPred-ANEL perform well on the Fruit Fly and Zebrafish datasets. As shown in Fig. 4a, LncPred-IEL trained on the main Human dataset produces the AUC scores of 0.8248 and 0.8999 for Fruit Fly and Zebrafish lncRNA prediction, respectively. LncPred-IEL trained on the main Mouse dataset achieves AUC scores of 0.9395 and 0.9841 for Zebrafish and Fruit Fly lncRNA prediction, respectively. As shown in Fig. 4b, LncPred-ANEL trained on the main Human dataset produces the AUC scores of 0.7928 and 0.8109 for Fruit Fly and Zebrafish lncRNA prediction, respectively. LncPred-ANEL trained on the main Mouse dataset achieves AUC scores of 0.9501 and 0.9410 for Fruit Fly and Zebrafish lncRNA prediction, respectively. That is, LncPred-IEL and LncPred-ANEL both can distinguish lncRNAs from coding RNAs in a cross-species manner, indicating both models have good generalization ability and can capture some common differences between lncRNAs and PCTs among the four species.

## Conclusion

LncRNA prediction is a very important topic in the field of bioinformatics. In this paper, we propose two lncRNA prediction models based on feature ensemble learning, namely LncPred-IEL and LncPred-ANEL. First, we encode the transcripts into feature vectors, we not only consider transcript-specified features but also adopt general sequence-derived features including spectrum profiles, mismatch profiles, reverse complement k-mer, pseudo nucleotide composition, and auto-cross covariance. We consider two strategies for feature ensemble, namely iterative ensemble learning (IEL) and attention network ensemble learning (ANEL). LncPred-IEL builds base predictors based on six types of features and employs a supervised iterative way to combine base predictors and build ensemble models. Extending our previous work, LncPred-ANEL employs an attention mechanism to ensemble six feature types, which places more attention on the features contributing more to prediction results. Experiments demonstrate that both LncPred-IEL and LncPred-ANEL can effectively separate positive instances and negative instances in the feature space. We also compare LncPred-IEL and LncPred-ANEL with other state-of-the-art models, and results show that LncPred-IEL and LncPred-ANEL produce overall better performances on evaluation metrics. LncPred-IEL and LncPred-ANEL’s ability of cross-species prediction are also tested on several datasets and obtain good results. In conclusion, LncPred-IEL and LncPred-ANEL are useful tools for lncRNA prediction and great complementary to experiments and traditional techniques.

## Methods

### Datasets

We collect two types of transcripts for experiments: lncRNAs and PCTs. We download the annotated lncRNAs and PCTs from GENCODE [35], which is a public repository containing annotations about the Human and Mouse genome performed by manual annotation, computational analysis, and experimental validation. We obtain lncRNAs and PCTs for Mouse (Release M21) and Human (Release 29) respectively. Because there are some redundant transcripts, we cluster transcripts and remove similar transcripts with a similarity threshold of 80% by using an open-source program called CD-HIT [36]. Then we take all lncRNAs as positive instances and sample the same number of PCTs as negative instances, and build our main datasets of Human and Mouse respectively.

We also adopt the Human (*Homo sapiens*), Mouse (*Mus musculus*), Fruit Fly (*Drosophila melanogaster*), and Zebrafish (*Danio rerio*) transcripts from CPPred datasets [14] for further analysis, which contains lncRNAs from Ensembl database [37] (Release 90) and PCTs from NCBI RefSeq [38] (Release 95).

The details about the datasets are demonstrated in Table 1. In the following sections, the main datasets are used to evaluate the proposed methods and compare different methods. CPPred datasets are used for parameter setting and cross-species lncRNA prediction.

### Feature extraction

In this section, we introduce six types of features to build lncRNA prediction models. In our study, we consider transcript-specified features, which are features specifically used for biological transcripts. Transcript-specified features are proved to be useful for lncRNA prediction by previous studies [8, 18, 22]. Besides, we also consider five types of general sequence-derived features, which are common statistical features of nucleotide sequences, containing spectrum profile, reverse complement k-mer profile, mismatch profile, pseudo nucleotide composition, and auto-cross covariance [39]. Details are described as follows. For the convenience of the feature description, we define a given transcript as

$$ S={N}_1{N}_2{N}_3\cdots {N}_L $$

where *L* is the total length of the transcript and *N*_*i*_ ∈ {A, G, C, U} denotes the *i* th nucleotide of the transcript.

#### Transcript-specified features

There are many transcript-specified features used in existing lncRNA prediction models. CPAT [22] adopts open reading frame (ORF) length, ORF coverage, Fickett score [40], and Hexamer score. CPC [8] and CPC2 [12] both utilize ORF integrity, isoelectric point (pI), Gravy, and Instability index. CPPred [14] employs the composition, transition, and distribution (CTD) features. The features are useful for lncRNA prediction, so we consider those transcript-specified features above when building our models. Details about these features are described below.

ORF length is the length of the first ORF in a transcript. ORF coverage is the ratio of the longest ORF length to the transcript length. Fickett [40] score is transformed from the nucleotide position frequencies and base composition of a transcript by a lookup table. Hexamer score [22] is calculated based on in-frame hexamer frequency of coding and non-coding transcripts, and a positive Hexamer score suggests a transcript is protein-coding. ORF integrity reflects whether the longest ORF starts with a start codon and ends with a stop codon. pI, Gravy, and Instability index are structure-related features. pI denotes the theoretical isoelectric point of the predicted peptide encoded by the given transcript. Gravy is the grand average of hydropathicity and Instability index reflects the stability of the predicted peptide [41].

The feature CTD [42] is a global transcript descriptor, which is composed of nucleotide composition, nucleotide transition, and nucleotide distribution. Nucleotide composition denotes the percentage composition of each nucleotide in the entire sequence. Nucleotide transition describes the percentage conversion frequency of four nucleotides between adjacent positions. Nucleotide distribution calculates the percentage conversion frequency of four nucleotides between five relative positions (0, 25, 50, 75, and 100%) along the transcript.

#### Spectrum profile

The spectrum profile, also known as *k*-mer, is a statistical ‘signature’ of the underlying sequence [43, 44]. *k*-mer describes the frequency of *k*-length contiguous subsequences. Given a sequence *S*, *k*-mer is defined as

$$ {f}_k^{spe}(S)=\left({c}_1,{c}_2,\cdots, {c}_{4^k}\right) $$

where *c*_*i*_ is the occurrence frequency of corresponding *k*-length contiguous subsequences.

#### Reverse complement k-mer (k-RevcKm) profile

This feature takes the reverse complement of the sequence into regard. Given a sequence *S*, the reverse complement *k*-length contiguous subsequences will be cut after generating *k*-mer, then the remaining *k*-length subsequences are extracted to create a feature vector called *k*-RevcKmer. For instance, if *k* = 2, there are 16 *k*-mers (‘AA’, ‘AC’, ‘AG’, ‘AT’, ‘CA’, ‘CC’, ‘CG’, ‘CT’, ‘GA’, ‘GC’, ‘GG’, ‘GT’, ‘TA’, ‘TC’, ‘TG’, ‘TT’), but by removing the reverse complementary *k*-mers, there are only 10 unique *k*-mers in the reverse complementary *k*-mer profile (‘AA’, ‘AC’, ‘AG’, ‘AT’, ‘CA’, ‘CC’, ‘CG’, ‘GA’, ‘GC’, ‘TA’). Detailed descriptions of this feature can be found in [45, 46].

#### Mismatch profile

The mismatch profile is similar to *k*-mer but has another parameter *m* (*m* < *k*) describing mismatch tolerance in the *k*-length contiguous subsequences. For instance, if *k* = 3 and *m* = 1, the symbol (3, 1) denotes a 3-length subsequence that has a maximum one mismatch. Assuming subsequence ‘ACG’ satisfies (3, 1), we need to consider 3 possible cases, ‘XCG’, ‘AXG’, and ‘ACX’ where ‘X’ in each case can be replaced by any nucleotide. (k, m)-mismatch profile is given by

$$ {f}_k^{mis}(S)=\left({\sum}_{j=0}^m{c}_{1j},{\sum}_{j=0}^m{c}_{2j},\cdots, {\sum}_{j=0}^m{c}_{4^kj}\right) $$

where *c*_*ij*_ denotes the occurrence frequency of the *i* th *k*-length contiguous subsequence with *j* mismatches, *i* = 1, 2, 3, ⋯, 4^*k*^ and *j* = 0, 1, 2, ⋯, *m*.

#### Pseudo nucleotide composition

In computational proteomics, the feature pseudo amino acid composition (PseAAC) is proposed by chou [47] to utilize the sequence-order information of protein sequences and has rapidly penetrated many areas of bioinformatics [48–50]. In our work, we take a variant form of PseAAC in the nucleotide research field called pseudo nucleotide composition as one of the features.

Pseudo nucleotide composition considers global sequence order information by the physicochemical properties of its constituent nucleotides [47]. In this study, various forms of pseudo nucleotide compositions are considered, including the basic feature pseudo dinucleotide composition (PseDNC) and four variants: parallel correlation pseudo dinucleotide composition (PC-PseDNC-General), parallel correlation pseudo trinucleotide composition (PC-PseTNC-General), series correlation pseudo dinucleotide composition (SC-PseDNC-General), and series correlation pseudo trinucleotide composition (SC-PseTNC-General).

#### Auto-cross covariance

Generated from the idea that a transcript can be viewed as a time sequence of the corresponding properties, auto-cross covariance [51] measures the correlation between properties of any two nucleotide residues and then transforms nucleotide sequences into vectors with fixed lengths. Auto-cross covariance has two components: auto-covariance (AC) and cross-covariance (CC).

AC measures the correlation of the same property between two nucleotides in the sequence, and the AC correlation of residue *i* between two nucleotides separated by a distance of *lag* can be calculated as

$$ \mathrm{AC}\left(i, lag\right)=\sum \limits_{j=1}^{L- lag}\left({S}_{i,j}-{\overline{S}}_i\right)\left({S}_{i,j+\mathrm{lag}}-{\overline{S}}_i\right)/\left(L- lag\right) $$

where *L* is the length of the sequence, *S*_*i*, *j*_ is the PSSM score of residue *i* at position *j*, ${\bar{S}}_i$ is the average PSSM score of residue *i* along the whole sequence. In this way, the dimensionality of AC is 4 × *LAG*, where *LAG* is the maximum of *lag*.

CC measures the correlation of two different properties between two nucleotides in the transcripts, and the CC correlation of residue *i*1 and residue *i*2 between two nucleotides separated by a distance of *lag* can be calculated as

$$ \mathrm{CC}\left(i1,i2, lag\right)=\sum \limits_{j=1}^{L- lag}\left({S}_{i1,j}-{\overline{S}}_{i1}\right)\left({S}_{i2,j+ lag}-{\overline{S}}_{i2}\right)/\left(L- lag\right) $$

where ${\bar{S}}_{i1}$ (${\bar{S}}_{i2}$) is the average PSSM score for residue *i*1 (*i*2). Because there are two residues in the formula, CC is not symmetrical, the total dimensionality of CC is 12 × *LAG*.

Different types of auto-cross covariance features combine AC and CC, such as dinucleotide auto-covariance (DAC), dinucleotide cross-covariance (DCC), trinucleotide auto-covariance (TAC), and trinucleotide cross-covariance (TCC). In this study, we adopt the combination of DAC and DCC (DACC) and the combination of TAC and TCC (TACC) [52].

### Iterative ensemble learning (IEL)

**Fig. 5:**
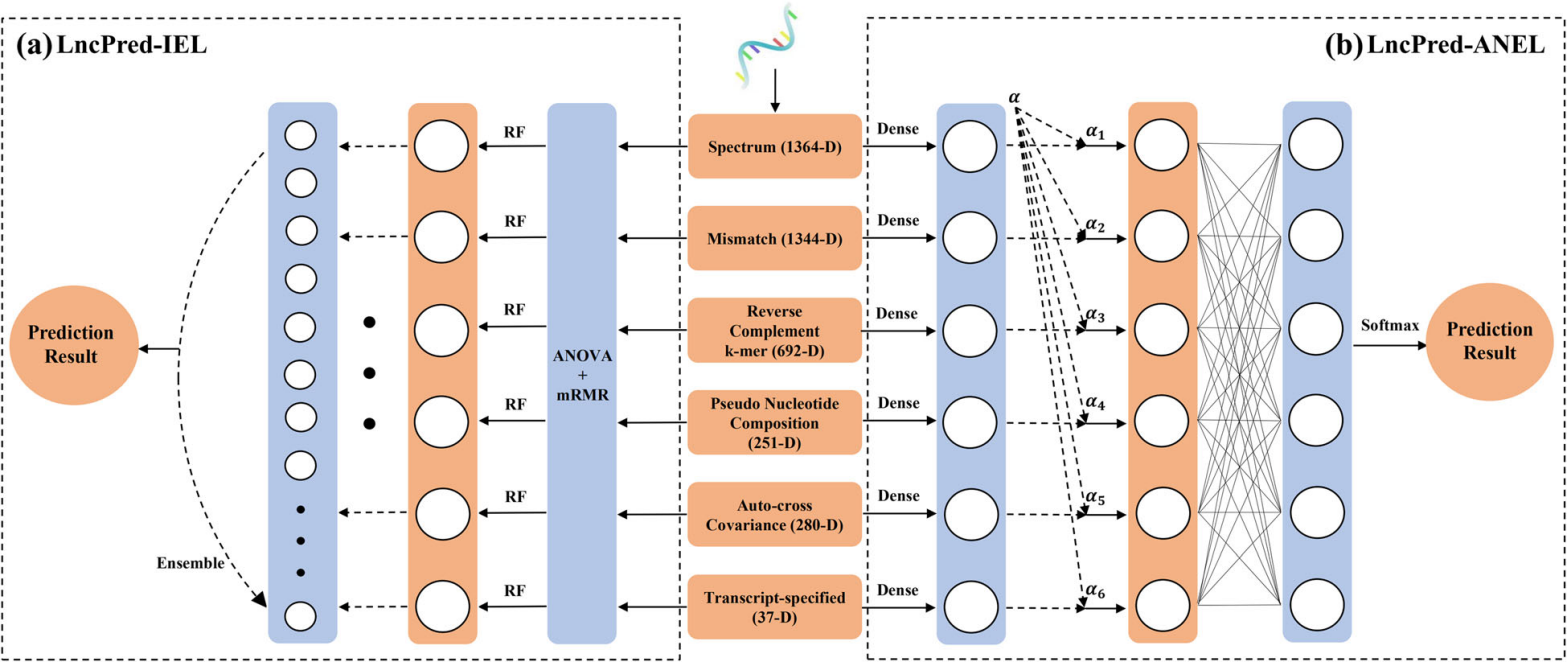
The workflow of LncPred-IEL (**a**) and LncPred-ANEL (**b**)

In this section, we introduce the iterative feature ensemble strategy to build the lncRNA prediction model, abbreviated as LncPred-IEL. The workflow of LncPred-IEL is shown in Fig. 5a.

First, we extract the six types of features in section feature extraction to encode the transcripts into feature vectors. As we can see in Table 2, some features have very high dimensions, which can result in feature redundancy and prediction noise. So, we adopt a two-step approach for feature selection to determine the most discriminative features subsets. The first step is to assess the importance of all features using Analysis of Variance (ANOVA) and sort all features according to their importance. ANOVA is a statistical method used to analyze the differences among group means in a sample [53]. Then we employ the minimal redundancy maximal relevance (mRMR) [54] to determine the optimal feature subsets. Therefore, we obtain six groups of optimal feature subsets for further analysis.

**Table 2:** Details of six types of features

	Feature		Dimensionality	Parameter
Transcript-specified	ORF length		1	No parameter
	ORF integrity		1	No parameter
	ORF coverage		1	No parameter
	Fickett score		1	No parameter
	Hexamer score		1	No parameter
	pI		1	No parameter
	Gravy		1	No parameter
	Instability index		1	No parameter
	CTD		30	No parameter
General sequence-derived	Spectrum profile	1-mer	4	No parameter
		2-mer	16	No parameter
		3-mer	64	No parameter
		4-mer	256	No parameter
		5-mer	1024	No parameter
	Mismatch profile	(3, m)-mismatch profile	64	m: the maximum mismatch
		(4, m)-mismatch profile	256	m: the maximum mismatch
		(5, m)-mismatch profile	1024	m: the maximum mismatch
	Reverse complement k-mer profile	1-RevcKmer	2	No parameter
		2-RevcKmer	10	No parameter
		3-RevcKmer	32	No parameter
		4-RevcKmer	136	No parameter
		5-RevcKmer	528	No parameter
	Pseudo nucleotide composition	PC-PseDNC-General	16 + λ	λ: the highest counted rank
		PC-PseTNC-General	64 + λ	λ: the highest counted rank
		SC-PseDNC-General	16 + 6 × λ	λ: the highest counted rank
		SC-PseTNC-General	64 + 12 × λ	λ: the highest counted rank
		PseDNC	16 + λ	λ: the highest counted rank
	Auto-cross covariance	DACC	36 × lag	lag: the distance between residues
		TACC	4 × lag	lag: the distance between residues

Second, we utilize the random forest (RF) classifier to construct the base predictors. RF is a commonly used machine learning algorithm for classification, regression, and other tasks [55]. We adopt the RF classifier to build base predictors because of its high efficiency and high accuracy. We use the Python package scikit-learn (v 0.20.3) [56] to implement RF classifiers. We build one base predictor on one optimal feature subset using the RF classifier. In this way, we build a total of six RF-based predictors using six groups of optimal feature subsets respectively.

Third, we use the iterative feature ensemble learning strategy to construct the final prediction model. The idea is originated from a layer-wise way of learning features in the deep neural network (DNN) [57]. We combine six base predictors to develop an ensemble model. In the stage of prediction, each of base predictors generates a score for a given sequence indicating the probability of the sequence being lncRNA. To integrate outputs from multiple base predictors, we adopt a novel nonlinear feature ensemble approach, which is described as follows. (i) For a given sequence, we combine the six probability scores generated by base predictors into a 6-dimensional feature vector. (ii) We build an RF-based ensemble model using the 6-dimensional feature vectors as input vectors, and the labels (lncRNA or PCT) of these sequences as outputs. (iii) We take the ensemble model as a new base predictor and add it to the set of base predictors. Iteratively, the ensemble model is generated from base predictors and then used as a new base predictor. (iv) The iteration process will continue until observing no performance improvement or reaching the maximum iteration round.

### Attention network ensemble learning (ANEL)

In this section, to better utilize and integrate the six types of features, we propose a novel attention network ensemble learning strategy to build the lncRNA prediction model as the extension of our previous work [32], abbreviated as LncPred-ANEL. The workflow of LncPred-ANEL is shown in Fig. 5b.

Inspired by the cognitive attention mechanism of the human brain, attention mechanism is designed in deep learning to selectively concentrate on a few relevant features [33]. The attention mechanism emerges as a result of the development of the neural network translation system in natural language processing (NLP) [33], and are widely used in other areas, such as bioinformatics [58], computer vision [59], speech processing [60], etc. Inspired by the hierarchical attention mechanism proposed by [61], we develop an attention network ensemble learning method called LncPred-ANEL.

First, there are six groups of features for a given sequence, and each group of features is respectively encoded into embeddings with the same dimensions using a fully-connected embedding layer. Let *f*_*i*_ denotes the *i* th embedding produced by the embedding layer, *i* = 1, 2, ⋯, 6. Second, an attention layer is designed to learn the attention embedding from six types of embeddings, the formulas are described as follows,

$$ {h}_i= ReLU\left({W}_w{f}_i+{b}_w\right) $$

$$ {\alpha}_i=\frac{\exp \left({h_i}^T{h}_w\right)}{\sum_i\mathit{\exp}\left({h}_i^T{h}_w\right)} $$

$$ {F}_{att}={\sum}_i{\alpha}_i{f}_i $$

Specifically, the embedding *f*_*i*_ is mapped into a hidden representation *h*_*i*_ using a nonlinear ReLU function, and *h*_*i*_ is normalized to obtain the attention weight *α*_*i*_. The attention embedding *F*_*att*_ is obtained by calculating the weighted sum of the six types of embeddings. *W*_*w*_, *b*_*w*_ and *h*_*w*_ are randomly initialized trainable parameters. Third, *F*_*att*_ is used as the input for a multilayer perceptron (MLP) to yield the prediction score.

We use PyTorch (v 1.5.0) [62] to implement LncPred-ANEL. We adopt the cross-entropy loss function, and choose the Adam optimizer with learning rate 5e-3, and set the batch size to 32. We use a dropout layer with a drop probability of 0.5 after the fully-connected feature embedding layer to prevent overfitting.

## Data Availability

The CPPred datasets used in this study are freely available at http://www.rnabinding.com/CPPred/. We download the main datasets from the GENCODE repository, the main datasets of Human and Mouse used in this study are available at https://www.gencodegenes.org/human/release_29.html and https://www.gencodegenes.org/mouse/release_M21.html.

## Abbreviations

LncPred-IEL: A lncRNA prediction method based on iterative ensemble learning strategy;
LncPred-ANEL: A lncRNA prediction method based on the attention network ensemble learning strategy;
5-CV: 5-fold cross-validation
ncRNA: Non-coding RNA
lncRNA: Long non-coding RNA
nt: Nucleotide
PCT: Protein-coding transcript
SVM: Support vector machine
RF: Random forest
LR: Logistic regression
RNN: Recurrent neural network
CNN: Convolutional neural network
DNN: Deep neural network
ORF: Open reading frame
pI: Isoelectric point
CTD: Composition, transition, and distribution
k-RevcKm: Reverse complement k-mer
PseDNC: Pseudo dinucleotide composition
PC-PseDNC-General: Correlation pseudo dinucleotide composition
PC-PseTNC-General: Parallel correlation pseudo trinucleotide composition
SC-PseDNC-General: Series correlation pseudo dinucleotide composition
SC-PseTNC-General: Series correlation pseudo trinucleotide composition
AC: Auto-covariance
CC: Cross-covariance
DAC: Dinucleotide auto-covariance
DCC: Dinucleotide cross-covariance
TAC: Trinucleotide auto-covariance
TCC: Trinucleotide cross-covariance
DACC: The combination of DAC and DCC
TACC: The combination of TAC and TCC
ANOVA: Analysis of Variance
mRMR: Minimal redundancy maximal relevance
NLP: Natural language processing
MLP: Multilayer perceptron
SN: Sensitivity
SP: Specificity
ACC: Accuracy
AUC: Area under ROC curve
ROC: Receiver operating characteristic
